# Minor Cannabinoids: Biosynthesis, Molecular Pharmacology and Potential Therapeutic Uses

**DOI:** 10.3389/fphar.2021.777804

**Published:** 2021-11-29

**Authors:** Kenneth B. Walsh, Amanda E. McKinney, Andrea E. Holmes

**Affiliations:** ^1^ Department of Pharmacology, Physiology and Neuroscience, School of Medicine, University of South Carolina, Columbia, SC, United States; ^2^ Institute for Human and Planetary Health, Crete, NE, United States; ^3^ School of Integrative Learning, Doane University, Crete, NE, United States; ^4^ Precision Plant Molecules, Denver, CO, United States

**Keywords:** *Cannabis sativa*, minor cannabinoids, TRP channel, endocannabinoids, therapeutics, CB1–CB2 cannabinoid receptors

## Abstract

The medicinal use of *Cannabis sativa L.* can be traced back thousands of years to ancient China and Egypt. While marijuana has recently shown promise in managing chronic pain and nausea, scientific investigation of cannabis has been restricted due its classification as a schedule 1 controlled substance. A major breakthrough in understanding the pharmacology of cannabis came with the isolation and characterization of the phytocannabinoids *trans*-Δ^9^-tetrahydrocannabinol (Δ^9^-THC) and cannabidiol (CBD). This was followed by the cloning of the cannabinoid CB1 and CB2 receptors in the 1990s and the subsequent discovery of the endocannabinoid system. In addition to the major phytocannabinoids, Δ^9^-THC and CBD, cannabis produces over 120 other cannabinoids that are referred to as minor and/or rare cannabinoids. These cannabinoids are produced in smaller amounts in the plant and are derived along with Δ^9^-THC and CBD from the parent cannabinoid cannabigerolic acid (CBGA). While our current knowledge of minor cannabinoid pharmacology is incomplete, studies demonstrate that they act as agonists and antagonists at multiple targets including CB1 and CB2 receptors, transient receptor potential (TRP) channels, peroxisome proliferator-activated receptors (PPARs), serotonin 5-HT_1a_ receptors and others. The resulting activation of multiple cell signaling pathways, combined with their putative synergistic activity, provides a mechanistic basis for their therapeutic actions. Initial clinical reports suggest that these cannabinoids may have potential benefits in the treatment of neuropathic pain, neurodegenerative diseases, epilepsy, cancer and skin disorders. This review focuses on the molecular pharmacology of the minor cannabinoids and highlights some important therapeutic uses of the compounds.

## Introduction

The marijuana plant has been grown and cultivated for medical, industrial and recreational uses throughout recorded history. Based on the physical characteristics of the plant, two main species of cannabis were originally described; *Cannabis indica* (short plant with broad leaves) and *Cannabis sativa* (tall plant with thin leaves) (Schultes, et al., 1974). However, numerous cannabis strains have been selected through breeding programs whose chemotaxonomic properties do not correlate with a *Cannabis indica* or *Cannabis sativa* lineage (Hillig and Mahlberg, 2004). More recently, the existence of only one species (*Cannabis sativa L.*), has been proposed (Small, 2015) with the strains categorized according to the content of the cannabinoids *trans*-Δ^9^-tetrahydrocannabinol (Δ^9^-THC) and cannabidiol (CBD). Δ^9^-THC dominant strains with low CBD content induce intoxicating, psychotropic effects including euphoria, enhancement of sensory perception and impairment in memory. In contrast, CBD dominant strains with low Δ^9^-THC content are considered to be nonpsychotropic.

Some of the earliest recorded medicinal uses of cannabis trace back to China and the pharmacopoeia of the Emperor Shen Nung (approximately 2500 BC), where the plant was indicated for the treatment of rheumatic pain, constipation, malaria and gynecological disorders (Russo, 2007; Pisanti and Bifulco, 2019). Along with China, cannabis medicine developed in India and then spread to Egypt, Greece and Rome. It was in Egypt that preparations of cannabis were first used in the treatment of glaucoma. By the 1800s extracts and tinctures of cannabis were recognized in the Western world for their relief of migraine headaches and their anti-emetic effects. In response to the perceived abuse of marijuana in the United States in the early 1900s, the Marijuana Tax Act was introduced which banned the sale and use of cannabis. This was followed in the 1970s by the classification of marijuana as a schedule 1 narcotic under the U.S. Controlled Substance Act. In Europe, the majority of countries have legalized the medical use of marijuana and decriminalized possession of small amounts of cannabis. However, the laws governing the use of cannabis can vary from one country to another with some countries having legalized only derivatives of the plant.

An important advancement in understanding the pharmacology of cannabis came with the isolation and structural determination of the phytocannabinoids CBD (Adams, et al., 1940a; Mechoulam and Shvo, 1963) and Δ^9^-THC (Gaoni and Mechoulam, 1971). Δ^9^-THC is the most abundant phytocannabinoid found in drug-type cannabis strains and the main psychotropic compound in the plant. In contrast, fiber-type strains have a higher content of CBD compared with Δ^9^-THC. CBD lacks psychotropic activity, but is reported to reduce the adverse effects (anxiety, psychosis, etc.) of Δ^9^-THC (Pennypacker and Romero-Sandoval, 2020). In addition to Δ^9^-THC and CBD, *Cannabis sativa L.* produces over 120 other phytocannabinoids as well as an abundance of related compounds including flavonoids, non-cannabinoid phenols, phenylpropanoids, fatty acids and terpenoids (Hanus, et al., 2016; Gülck and Möller, 2020). Phytocannabinoids are meroterpenoids (21- and 22-carbon terpenophenolic compounds with an alkyl side chain) produced in the plant’s glandular trichomes (Hanus, et al., 2016; Gülck and Möller, 2020). Cannabigerolic acid (CBGA), synthesized in cannabis from geranyl pyrophosphate and olivetolic acid, represents the parent cannabinoid from which the acidic and neutral minor cannabinoids are derived. In general, Δ^9^-THC and CBD are considered the major phytocannabinoids, while other phytocannabinoids, present in smaller amounts in the plant, are referred to as minor (or rare) cannabinoids.

More than 230 million people worldwide consume marijuana making it the most commonly used illicit substance (World Health Organization, 2016). In recent years, cannabis has become more accessible in the United States and Europe due to its legalization for medicinal and recreational purposes. While administered for a large number of medical conditions including nausea, anorexia, glaucoma, and muscle spasms, observational studies and user surveys indicate that pain management is the most common indication for the use of cannabis (Romero-Sandoval, et al., 2018). For this purpose, medical cannabis can be smoked or vaporized (using the floral buds of the plant), applied *via* oromucosal spray preparations [cannabis extract in Nabiximols (Sativex^®^)] or swallowed in capsule form as Nabilone (Cesamet^®^, synthetic cannabinoid) and Dronabinol (Marinol^®^, synthetic Δ^9^-THC). Anecdotal evidence indicates that the combination of the phytocannabinoids, terpenoids and other phytochemicals present in the whole cannabis plant provides a greater efficacy (called the “entourage effect”) in treating chronic pain when compared to oral cannabinoid formulations (Russo, 2011). However, definitive experimental data supporting this synergistic effect are currently lacking (Santiago, et al., 2019; Finlay, et al., 2020). Finally, in addition to its own direct anti-nociceptive effects, medical cannabis may have opioid drug-sparing actions: thus allowing lower doses of opioids to be used for pain relief (Khan, et al., 2019).

This review provides a brief description of the biosynthesis of the phytocannabinoids and an overview of the endocannabinoid system. This is followed by a discussion of the molecular pharmacology and potential therapeutic uses of the minor cannabinoids. Readers desiring information on Δ^9^-THC, CBD or synthetic cannabinoids are directed to these recent reviews (Banister, et al., 2019; de Almeida and Devi, 2020; Alves, et al., 2020; Walsh and Andersen, 2020).

## Biosynthesis of Phytocannabinoids

**FIGURE 1:**
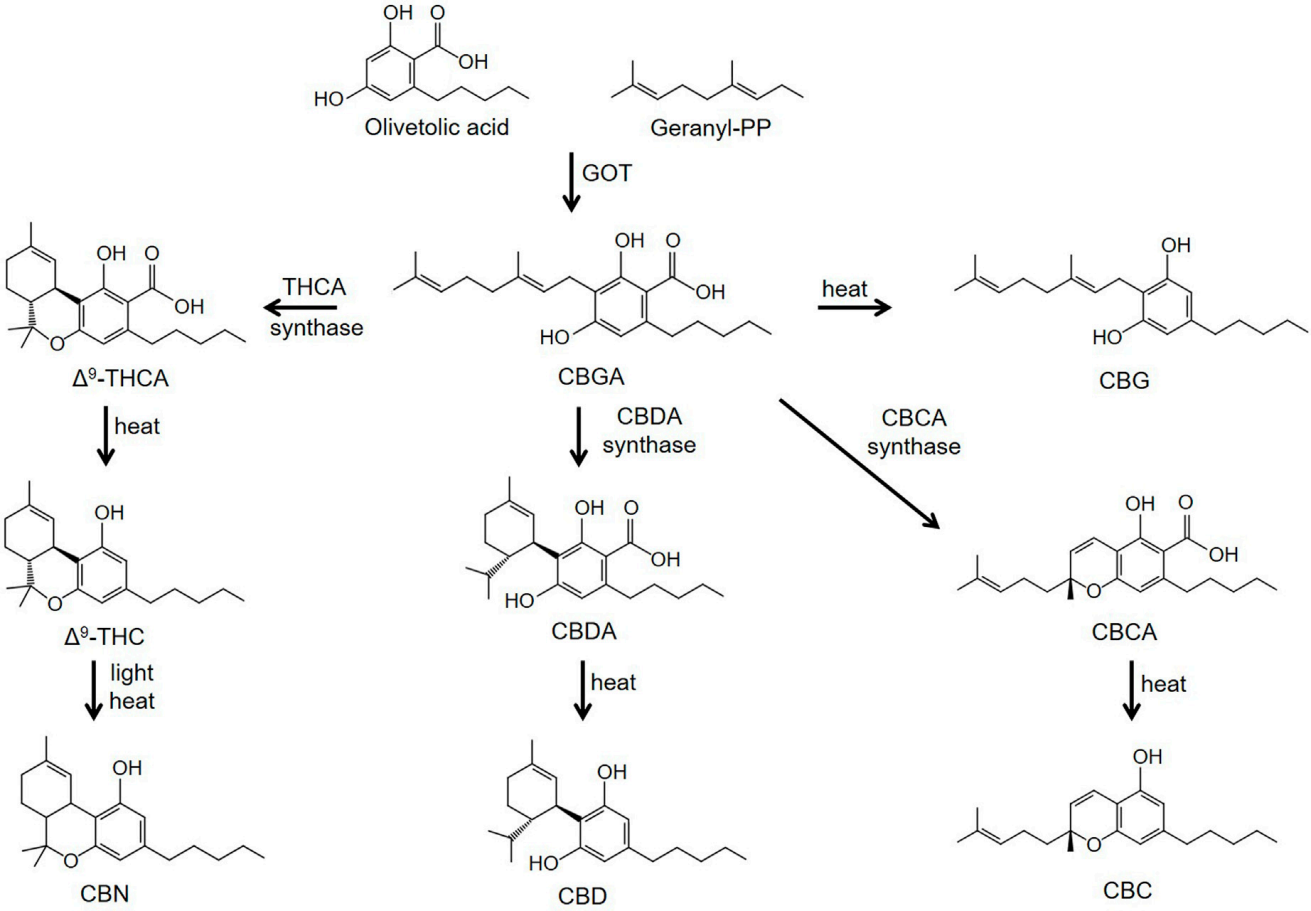
Biosynthesis pathways of phytocannabinoids. Abbreviations: CBN, cannabinol; CBC, cannabichromene; CBD, cannabidiol; CBG, cannabigerol; CBDA, cannabidioloic acid; CBGA, cannabigerolic acid; CBCA, cannabichromenic acid; GOT, olivetolate geranyltransferase; Δ^9^-THCA, Δ^9^-tetrahydrocannabinolic acid; Δ^9^-THC, Δ^9^-tetrahydrocannabinol.

Phytocannabinoids are meroterpenoids consisting of 21 or 22 carbon atoms that usually contain a propyl or pentyl side chain (Hanus, et al., 2016; Gülck and Möller, 2020). In *Cannabis sativa L.* the phytocannabinoids and terpenes are synthesized and stored in the glandular trichomes that are found in highest density in the female flowers of the plant (Hanus, et al., 2016; Gülck and Möller, 2020). The synthesis of the cannabinoids involves two pathways located in two separate sites within the glandular trichomes. In the first pathway olivetolic acid (OA) is produced in the cytosol of the gland cells from hexanoic acid. In the second, geranyl diphosphate (GPP) is generated in the plastidial organelles *via* the mevalonate-dependent isoprenoid (MEP) pathway. CBGA, the precursor of phytocannabinoids containing a pentyl side chain, is then synthesized from the GPP prenylation of olivetolic acid; a reaction catalyzed by olivetolate geranyltransferase (GOT) (Figure 1). Synthesis of tetrahydrocannabinolic acid (THCA), cannabidioloic acid (CBDA), and cannabichromenic acid (CBCA) proceeds through the appropriate oxidocyclases, THCA synthase, CBDA synthase and CBCA synthase, respectively (Figure 1). As described below, the neutral phytocannabinoids are then derived from the acidic forms through non-enzymatic decarboxylation during exposure to heat or light. Cannabigerovarin acid (CBGVA), the precursor to the variant cannabinoids (propyl side chain), is synthesized from divarinolic acid through geranyltransferase (Hanus, et al., 2016).

Various methodologies including extraction/isolation, semi-synthesis or full synthesis and microbial engineering (*E. coli*, algae, yeast etc.,) are being utilized to obtain minor cannabinoids. Selective cannabis crossbreeding to enhance or decrease certain cannabinoids or terpenes is a common practice. For example, selective breeding has been used to yield cannabis varieties rich in CBG, CBC, THCV and CBDV (de Meijer and Hammond, 2005; de Meijer, et al., 2009). In a major advancement in the field, Luo et al. (2019) successfully introduced the MEP, GPP and hexanoic pathways along with THCA and CBDA synthases in yeast (*Saccharomyces cerevisiae*); establishing a platform for the large-scale fermentation of natural minor cannabinoids. Interestingly, unnatural cannabinoids with tailored alkyl side chains were produced by feeding different fatty acid precursors to the yeast (Luo, et al., 2019). Since the length and chemistry of the alkyl side chain modulates the affinity of the cannabinoids for the CB1 and CB2 receptors (Martin, et al., 1999), this platform could provide a novel method for discovering new and novel cannabinoid receptor specific agonists and antagonists.

## The Endocannabinoid System

### Cannabinoid CB1 and CB2 Receptors

**FIGURE 2:**
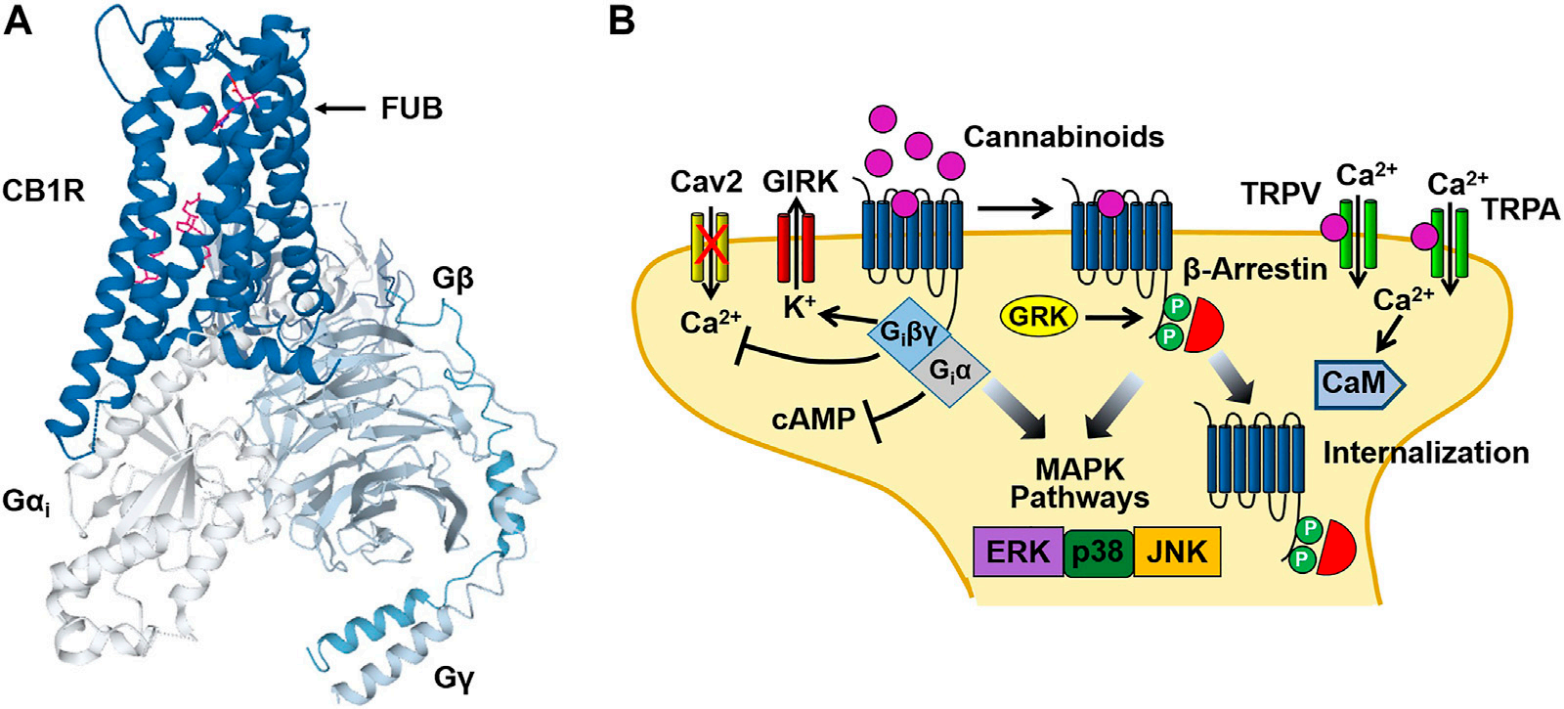
Cannabinoid CB1 receptor structure and signaling. **(A)** Structural model of the CB1 receptor (CB1R)-G_i_ protein complex obtained from cryoelectron microscopy. The binding site for the synthetic cannabinoid MDMB-FUBINACA (FUB) is indicated by the magenta structure. The CB1-G_i_ receptor complex structure was obtained from the Protein Data Bank (code 6N4B). **(B)** Binding of cannabinoids to the neuronal CB1 receptor stimulates both neuronal G_i_/G_o_ and β-arrestin signaling pathways leading to an inhibition of adenylyl cyclase, activation of G protein-gated inward rectifier K^+^ (GIRK) channels and receptor internalization. In addition, activation of transient receptor potential vanilloid (TRPV) and ankyrin (TRPA) channels by cannabinoids causes Ca^2+^ influx that activates Ca^2+^-sensitive enzymes such as Ca^2+^/calmodulin-dependent protein kinase (CaM). Figure 2B was adapted from Walsh and Andersen (Walsh and Andersen, 2020).

Cannabinoid investigators initially hypothesized that Δ^9^-THC might act by disturbing cell membranes due to its lipophilic properties. However, binding assays obtained using the radio-labeled synthetic cannabinoid CP-55,940 [^3^(H)-CP-55,940], identified selective, high affinity binding sites for the compound in rat brain preparations (Devane, et al., 1988). This finding led to the cloning of the cannabinoid type 1 (CB1) (Matsuda, et al., 1990) and type 2 (CB2) (Munro, et al., 1993) receptors in the early 1990s. Both the CB1 and CB2 receptors are members of the G protein-coupled receptor (GPCR) super-family of proteins. CB1 receptors are primarily localized to presynaptic nerve terminals in the central and peripheral nervous system. Tissues expressing high levels of the CB1 receptor include the amygdala, hippocampus, cerebral cortex, cerebellum and spinal column (Herkenham, et al., 1990; Tsou, et al., 1998). In contrast, CB2 receptors are found in the cells of the immune system and in astrocytes and microglia of the CNS (Munro, et al., 1993; Galiégue, et al., 1995; Stella, 2010). As is the case with other Class A GPCRs, cannabinoid receptors contain seven transmembrane domains (TM1-7) with intracellular (ICLs) and extracellular loops (ECLs), an N-terminal ECL and an intracellular domain that interacts with pertussis toxin-sensitive G proteins (G_i_/G_o_) (Figure 2). Using computational modeling with the CB1 receptor crystal structure, Hua et al. (2017) predicted that Δ^9^-THC interacts with the ECL2 and TM3, TM6 and TM7. Binding of cannabinoids is postulated to activate a toggle switch in the CB1 receptor (consisting of residues F200 and W356 in the TM3/TM6 binding pocket) that results in G_i_/G_o_ protein interaction (Hua, et al., 2017). The strong interaction of the indazole ring of the synthetic cannabinoid MDMB-FUBINACA (FUB) with the toggle switch stabilizes the active conformation of the receptor and brings about the high efficacy of this ligand (Kumar et al., 2019) (Figure 2). In contrast, (Kumar et al., 2019) suggested that the lack of toggle switch interaction by Δ^9^-THC may account for its partial agonist activity. Although cannabinoid receptors primarily couple to G_i_/G_o_, they can also stimulate G_s_ and G_q_ proteins under certain conditions (Glass and Northup, 1999; Lauckner, et al., 2005).

Binding of Δ^9^-THC and synthetic cannabinoids (WIN 55,212-2, CP 55,940, etc.,) to the CB1 and CB2 receptors causes the dissociation of the βγ subunits of the G protein heterotrimer from the α subunit (G_i_α) (Figure 2). G_i_α inhibits adenylyl cyclase resulting in a decrease in intracellular levels of cAMP (Howlett, et al., 1986). In contrast, G_i_βγ inhibits the opening of voltage-gated Ca^2+^ channels (N and P/Q type) while activating G protein-gated inward rectifier K^+^ (GIRK) channels (Mackie, et al., 1995; Guo and Ikeda, 2004). In addition to GIRK channels, cannabinoid binding also couples to other K^+^ channels including M-type and A-type channels in cultured neurons (Schlicker and Kathmann, 2001). Together, these cannabinoid actions bring about an acute inhibition of synaptic neurotransmitter release and dampens neuronal excitability (Shen, et al., 1996; Vaughan, et al., 2000). These signaling effects are followed by receptor phosphorylation [by G protein receptor kinase (GRK)] that recruits β-arrestin1 (βarr1) and β-arrestin2 (βarr2) to the receptor and results in CB1 receptor desensitization and internalization (Figure 2) (Jin, et al., 1999; Ahn, et al., 2013). Both G_i_ and β-arrestin can also stimulate mitogen-activated protein kinases (MAPKs), including the extracellular signal-regulated kinases (ERK1/2), bringing about additional cellular effects (Bouaboula, et al., 1995; Galve-Roperh, et al., 2002; Derkinderen, et al., 2003).

### Endocannabinoids

Following the discovery of the CB1 and CB2 receptors, the endogenous cannabinoids (or endocannabinoids) anandamide [N-arachidonoylethanolamine (AEA)] and 2-arachidonoylglycerol (2-AG) were isolated (Devane, et al., 1992; Mechoulam, et al., 1995; Sugiura, et al., 1995). These endocannabinoids are synthesized from the cell membrane lipids N-arachidonoyl phosphatidyl ethanol (NAPE) (for AEA) and phosphatidyl inositol bis-phosphate (PIP2) (for 2-AG). Unlike the continuous cellular synthesis and storage of neurotransmitters and neuropeptides, AEA and 2-AG are produced through “on demand” cleavage of NAPE and PIP2. This provides for a temporal- and localization-dependent release of the endocannabinoids (Lu and Mackie, 2016). The actions of AEA and 2-AG are terminated following their cellular uptake and degradation by intracellular hydroxylase [fatty acid amide hydrolase (FAAH)] (for AEA) and lipase enzymes (monoacylglycerol lipase) (for 2-AG). Therefore, drugs that inhibit the cellular uptake of AEA and 2-AG or prevent their enzymatic degradation should result in a potentiation of endocannabinoid action. In addition to AEA and 2-AG, other putative endocannabinoids include N-arachidonoyldopamine (NADA), 2-arachidonoylglycerylether (noladin ether), N-oleoylethanolamine (OEA) and palmitoylethanolamide (PEA) (Tan, et al., 2006).

Endocannabinoids bring about their pharmacological effects through a number of mechanisms. Early studies demonstrated that while 2-AG acts as a full agonist at the CB1 and CB2 receptors, both AEA and Δ^9^-THC function as partial agonists when compared with the full cannabinoid agonist WIN 55,212-2 (Gonsiorek, et al., 2000; Luk, et al., 2004). Endocannabinoids can also act at receptor sites (“off targets”) other than the CB1 and CB2 receptors. Transient receptor potential (TRP) channels are a superfamily of inotropic channels that are activated by thermal, physical and electrochemical stimuli. The TRP channels are subdivided into several families including the vanilloid (TRPV), ankyrin (TRPA) and melastatin (TRPM) channels. Both AEA and 2-AG bind to and activate TRPV1 channels causing cell membrane potential depolarization and Ca^2+^ influx (Zygmunt, et al., 1999; Smart, et al., 2000) (Figure 2). AEA can also modulate the activity of TRPA1 and TRPM8 channels (De Petrocellis, et al., 2007; De Petrocellis, et al., 2012). In addition to the TRP channels, a number of other “off targets” for endocannabinoids have been identified. This includes the de-orphanized G protein-coupled receptors 18 (GPR18) and 55 (GPR55), as well as Peroxisome Proliferator-activated Receptors (PPARs). GPR18 and GPR55 are proposed to regulate acute and chronic pain pathways and are activated by the endogenous ligands N-arachidonoylglycine (NAgly) and lysophophatidylinositol (LPI), respectively (Kohno, et al., 2006; Henstidge, et al., 2009). Binding of endocannabinoids to these receptors stimulates cell signaling events through Gα_i_ (GPR18), Gα_12_ and Gα_12/13_ (GPR55). PPARs are members of the nuclear hormone receptor family of proteins that function as ligand-inducible transcription factors. A number of endocannabinoids including AEA and 2-AG, as well as the phytocannabinoids Δ^9^-THC and CBD, are agonists at the PPARs (O'Sullivan, 2007).

## Minor Cannabinoid Pharmacology and Therapeutics

Minor cannabinoids are divided into neutral, acidic and varinic phytocannabinoids. Minor cannabinoids include cannabinol (CBN), cannabichromene (CBC), cannabigerol (CBG), cannabidioloic acid (CBDA), cannabigerolic acid (CBGA), tetrahydrocannabinolic acid (THCA), cannabinolic acid (CBNA), cannabidivarin (CBDV), tetrahydrocannabivarin (THCV), cannabigerovarin (CBGV), cannabichromevarin (CBCV), and others (Hanus, et al., 2016; Gülck and Möller, 2020). Cannabinoids appear naturally in the cannabis plant in their acidic forms and are thought to confer antioxidant and defense mechanisms (insecticidal, antimicrobial, etc.,) to the plant. Acidic cannabinoids undergo decarboxylation during heating and are converted to the corresponding neutral cannabinoids (Figure 1 **)**. For example, THCA is converted to Δ^9^-THC when cannabis is smoked or vaporized. Some decarboxylation also occurs with passage of time at room temperature and during exposure to light. Cannabis products intended to contain the acidic forms of cannabinoids nearly universally also contain low levels of cannabinoids in their neutral forms. The varinic cannabinoids are considered rare but are now emerging as new targets of selective breeding. Varin compounds such as CBDV and THCV contain two fewer carbon atoms than their non-varin counterparts (CBD and Δ^9^-THC) endowing these cannabinoids with unique pharmacological properties (see below).

As described for the endocannabinoids, the overall pharmacological action of the minor cannabinoids often results from binding at both cannabinoid and “off target” receptors. This combination of receptor-mediated actions makes them well suited as multi-target therapeutic agents. While a number of minor cannabinoids including CBN and THCV bind to the CB1 receptor, they have significantly less binding activity when compared with Δ^9^-THC (Rhee, et al., 1997; Zagzoog, et al., 2020). To date, none of the minor cannabinoids have been clinically demonstrated to act as psychotropic drugs. The reported potencies of the minor cannabinoids at the CB1 and CB2 receptors and TRP channels are summarized in Table 1 and discussed in the sections below.

**TABLE 1:** Pharmacology of the minor cannabinoids.

Receptor/Cell	EC_50_/IC_50_ (µM)^a^									References
	Assay	CBN	C-BC	CBG	CBDA	CBGA	THCA	CBDV	THCV	
CB1/COS-7^b^	cAMP inhibition	0.12								Rhee, et al. (1997)
CB1/HEK293^c^	cAMP inhibition						>10			McPartland, et al. (2017)
CB1/HEK293^c^	cAMP inhibition^k^				1	1		1		Navarro, et al. (2020)
CB1/CHO^d^	cAMP -inhibition^l^		0.19	0.12	0.03		>10	>10	0.26	Zagzoog, et al. (2020)
CB1/cerebellum^e^	GTPγS binding								0.03	Dennis, et al. (2008)
CB1/HEK293^c^	GTPγS binding	0.31		>10		0.18	>10	>10	>10	Husni, et al. (2014)
CB1/vas deferens^f^	EECs								>10	Thomas, et al. (2005)
CB2/COS-7^b^	cAMP inhibition	0.290								Rhee, et al. (1997)
CB2/HEK293^c^	cAMP inhibition				0.1			>1		Navarro, et al. (2020)
CB2/CHO^d^	cAMP inhibition		0.007	0.13	0.14		1.8	0.005	0.28	Zagzoog, et al. (2020)
CB2/CHO^d^	cAMP inhibition								0.038	Bolognini, et al. (2010)
CB2/HEK293^c^	GTPγS binding	0.29		1.21			>10	0.003	>10	Husni, et al. (2014)
CB2/AtT20^g^	membrane potential		>3							Udoh, et al. (2019)
TRPV1/HEK293^h^	Ca^2+^ signal	6.2	24.2	1.3	19.7	21.0		3.6	1.5	De Petrocellis, et al. (2011)
TRPV2/HEK29^h^	Ca^2+^ signal	19.0		1.7			18.4	7.3	4.1	De Petrocellis, et al. (2011)
TRPV3/HEK29^h^	Ca^2+^ signal					12.6			3.8	De Petrocellis, et al. (2012)
TRPV4/HEK29^h^	Ca^2+^ signal	16.1		5.1		28.8		0.9	6.4	De Petrocellis, et al. (2012)
TRPA1/HEK293^i^	Ca^2+^ signal	0.18	0.09	0.7	5.3	8.4	2.7	0.42	1.5	De Petrocellis, et al. (2011)
TRPM8/HEK293^j^	Ca^2+^ signal	0.21	40.7	0.14	0.9	1.31	0.14	0.9	0.87	De Petrocellis, et al. (2008)

a Cannabinoid potencies as agonists (EC_50_) and antagonists (IC_50_).

b African green monkey kidney (COS-7) cells expressing either the rat CB1 or CB2 receptor.

c HEK293 cells expressing either the human CB1 or CB2 receptor.

d Chinese hamster ovary (CHO) cells expressing either the human CB1 or CB2 receptor.

e Antagonism of WIN-55,212-2 stimulation of (^35^S)GTPγS binding in rodent brain cerebellum.

f Inhibition of electrically-evoked contractions (EECs) of mouse vas deferens (IC_50_).

g Membrane potential measured using a fluorescent dye in pituitary AtT20 cells expressing the CB2 receptor.

h Ca^2+^ fluorescence measured in HEK293 cells expressing the rat TRPV1-TRPV4 channels.

i Ca^2+^ fluorescence measured in HEK293 cells expressing the rat TRPA1 channel.

j Ca^2+^ fluorescence measured in HEK293 cells expressing the rat TRPV1-TRPV4 channels (IC_50_ against icilin).

k EC_50_ values are not given by Navarro et al., 2020. Listed values were estimated from the displayed concentration versus response curves.

l CHO cells treated for 90 min with cannabinoids.

### Neutral Cannabinoids

#### Cannabinol

CBN was originally isolated from Indian hemp in 1896 making it the first phytocannabinoid identified in cannabis (Wood, et al., 1886). The structural determination and total synthesis of CBN was carried out by Adams and colleagues in the 1940s (Adams, et al., 1940b). CBN is not synthesized in the cannabis plant, but is derived during the degradation of Δ^9^-THC. Even under ideal storage conditions, exposure to UV light and/or heat over time results in the conversion of Δ^9^-THC to CBN. Using ^3^(H)-labelled synthetic cannabinoid (e.g., HU-243, CP-55,490) displacement assays it was determined that CBN has low binding affinities for the CB1 and CB2 receptors when compared with Δ^9^-THC (Rhee, et al., 1997; Mahadevan, et al., 2000; Rosenthaler, et al., 2014). In addition, CBN is less potent than Δ^9^-THC in CB1 receptor-mediated inhibition of adenylyl cyclase, but displays equal potency in CB2 receptor-mediated inhibition (Rhee, et al., 1997). CBN is an agonist at TRPV1, TRPV2, TRPV3 and TRPV4 channels stimulating cell Ca^2+^ influx with the activation of Ca^2+^-dependent pathways (De Petrocellis, et al., 2011). It is also a potent and efficacious agonist of the TRPA1 channel. In addition, CBN acts as a potent antagonist of icilin activation of the TRPM8 channel (De Petrocellis, et al., 2011).

CBN has been identified as a potential analgesic and anti-inflammatory agent. CBN isolate has been reported to relieve chronic muscle pain disorders such as temporomandibular disorders and fibromyalgia in a rat model of myofascial pain (Wong and Cairns, 2019). For example, CBN (1 mg/ml) reduces mechanical sensitivity induced by intramuscular injection of nerve growth factor in the masseter muscle (Wong and Cairns, 2019). While CBN is not as widely recognized as CBD and Δ^9^-THC for its anti-inflammatory properties, it may have therapeutic benefits in treating allergic airway diseases. CBN attenuates the production of interleukins 2, 4, 5, 13 and decreases allergen mucus production in OVA-sensitized and challenged A/J mice (Jan, et al., 2003). CBN and Δ^9^-THC (but not CBD) can also be used to treat glaucoma since they prevent inflammation that causes elevated intraocular pressure (ElSohly, et al., 1981). In addition, preliminary data indicate that CBN decreases cell damage and acts as an antioxidant in a cell culture model of Huntington’s disease (Aiken, et al., 2004).

CBN also shows promise as an antibacterial agent and an appetite stimulant. As with other cannabinoids (e.g., CBC and CBG), CBN has been found to be highly efficacious against multiple antibiotic-resistant bacteria, including methicillin-resistant Staphylococcus aureus (MRSA), making it a potentially viable treatment for staph infections (Appendino, et al., 2008). CBN also stimulates hyperphagia and increases food consumption and feeding time in rats (Farrimond et al., 2012a; Farrimond, et al., 2012b). Although CBN is not as potent an appetite stimulant as Δ^9^-THC, CBN administration is not associated with the psychotropic effects of Δ^9^-THC. Thus, CBN represents a non-intoxicating alternative to Δ^9^-THC as an appetite stimulant.

CBN-rich products are advertised for promoting sleep or relaxation without the impairment caused by Δ^9^-THC. Since CBN is a degradation product of Δ^9^-THC it is found in greater quantities in aged cannabis preparations. For this reason it is marketed as “the sleepy cannabinoid in old weed.” However, laboratory results obtained from sleep studies with CBN have been equivocal. In mice, CBN was reported to increase barbiturate-induced sleep time in one study (Yoshida, et al., 1995), while having no effects on sleep in another (Chesner, et al., 1974). When administered along with Δ^9^-THC in rats, CBN produces greater sedation compared with either cannabinoid alone (Fernandes, et al., 1974; Takahashi and Karniol, 1975). In one clinical study involving a small number of participants, the combination of Δ^9^-THC and CBN caused greater drowsiness than with Δ^9^-THC used alone (Karniol, et al., 1975). However, in a recent review of the CBN literature, Corroon (2021) found little evidence supporting a sleep promoting effect of CBN. Therefore, controlled studies are warranted to substantiate sleep-related claims of CBN containing products.

#### Cannabichromene

CBC is one of the most abundant minor cannabinoids found in cannabis. The structure of CBC was first determined using NMR spectroscopy by Gaoni and Mechoulam (Gaoni and Mechoulam, 1966). Although cannabinoid receptor studies using CBC are limited, the cannabinoid was initially identified as partial CB2 receptor agonist (Rosenthaler, et al., 2014). This was supported by experiments using a “hyperpolarization assay” with pituitary AtT20 cells in which CBC was found to be selective in stimulating the CB2 receptor over the CB1 receptor (Udoh, et al., 2019). In this same study, CBC was more potent and efficacious than Δ^9^-THC in causing cell hyperpolarization *via* the CB2 receptor. In contrast to these results, CBC was shown in a recent paper to display similar affinities for the CB1 and CB2 receptors and to cause both CB1 and CB2 receptor-mediated decreases in cellular cAMP levels (Zagzoog, et al., 2020). As is the case for CBN, CBC is a potent activator of the TRPA1 channel (De Petrocellis, et al., 2011). In addition, it activates TRPV3 and TRPV4 channels when applied at micromolar and submicromolar concentrations (De Petrocellis, et al., 2011). Other proposed sites of action of CBN are discussed below.

Anti-inflammatory effects of CBC were first reported in the 1980s using a rat model of edema. High doses of CBC were more efficacious than the nonsteroidal anti-inflammatory drug (NSAID) phenylbutazone in carrageenan-induced paw edema (Turner and ElSohly, 1981). CBC has been shown to reduce pain and inflammation associated with osteoarthritis in rats without the negative side effects of NSAIDs (Maione, et al., 2011). In addition, CBC attenuates lipopolysaccharide (LPS)-induced increases in nitric oxide levels in an *in vitro* model of colitis (Romano, et al., 2013) and reduces inflammation-induced GI motility (Izzo, et al., 2012). CBC also displays a modest anti-nociceptive effect in the mouse tail-withdrawal assay (Maione, et al., 2011; Zagzoog, et al., 2020). CBC regulates a number of cellular pathways involved in anti-nociception that include the stimulation of adenosine A1 receptors, CB1 receptors and TRPA1 channels (De Petrocellis, et al., 2011; Maione, et al., 2011). In addition, CBC has been proposed to inhibit AEA (anandamide) reuptake; thus potentiating the physiological effects of AEA (De Petrocellis, et al., 2011). Like other synergistic actions of cannabinoids, CBC has a greater anti-inflammatory response when combined with Δ^9^-THC than when either cannabinoid is used alone (DeLong, et al., 2010).

The anti-inflammatory actions of CBC may be important in its ability to function as a neuroprotective drug. CBC increases the viability of neural stem progenitor cells (NSPCs) *in vitro* through an ERK dependent mechanism (Shinjyo and Di Marzo, 2013). In addition, CBC inhibits astroglial differentiation of the NSPCs (Shinjyo and Di Marzo, 2013). NSPCs are modulated by surrounding microglial cells, brain immune cells, and astrocytes, which produce both pro- and anti-inflammatory factors. The potential anti-inflammatory and neuroprotective effects of CBC may occur through its suppression of reactive astrocytes (Covelo, et al., 2021). CBC inhibition of NSPCs differentiation into astrocytes may therefore offer a protective effect against neuro-inflammation, Alzheimer’s disease, and hepatic encephalopathy (Covelo, et al., 2021).

Δ^9^-THC possesses anti-tumor properties and is used for treating several different forms of cancer (Fraguas-Sánchez and Torres-Suárez, 2018). However, the psychotropic qualities of Δ^9^-THC limit its use as a chemotherapy agent. CBC may be beneficial in cancer treatment due to its ability to increase blood levels of AEA (see above). AEA has been shown to inhibit breast cancer cell proliferation and induce death of colon cancer cells (De Petrocellis, et al., 1998; Patsos, et al., 2005). CBC was also shown to inhibit cell migration and disrupt the cell cytoskeleton in an *in vitro* model of urothelial cancer (Anis, et al., 2021). In one study that examined the anti-tumor effects of several minor cannabinoids, only CBG was more potent than CBC at inhibiting the growth of several cancer cell lines (Ligresti, et al., 2006).

#### Cannabigerol

CBG is produced *via* decarboxylation of CBGA, the precursor molecule of the Δ^9^-THC and CBD branches of the cannabis synthesis pathway (Figure 1). Results obtained using cAMP assays revealed that CBG displays weak partial agonist activity at the CB1 and CB2 receptors (see Table 1) (Husni, et al., 2014; Navarro, et al., 2020; Zagzoog, et al., 2020). In CHO cells expressing both CB1 and CB2 receptors, CBG binds to the receptors with K_i_s in the low micromolar range (Navarro, et al., 2018). Of special note, in this same study CBG was found to compete with ^3^(H)-CP-55,490 for binding to the CB1 receptor, but not ^3^(H)-WIN-55,212-2 (Navarro, et al., 2018). This suggests the CBG and CP-55,490 (but not WIN-55,212-2) bind to the same orthosteric site on the receptor. CBG activates TRPV1, TRPV2, TRPV3, TRPV4 and TRPA1 channels at low micromolar concentrations (De Petrocellis, et al., 2011). CBG has also been shown to act through other off-target sites including the 5-HT_1a_ receptor and the α_2_-adrenergic receptor. For example, CBG competitively antagonizes the ability of the 5-HT_1a_ agonist 8-hydroxy-2-(di-n-propylamino)tetralin (8-OH-DPAT) to stimulate (^35^S)GTPγS binding in rat brain membranes (Cascio, et al., 2010). Furthermore, CBG inhibits electrically-induced contractions of the vas deferens and stimulates (^35^S)GTPγS binding in rat brain membranes through agonist activity at the α_2_-adrenergic receptor (Cascio, et al., 2010). CBG, along with acidic cannabinoids THCA and CBDA, also binds to and activates PPARγ (D'Aniello, et al., 2019) (see below).

As with other minor cannabinoids, CBG may reduce the severity of inflammatory diseases and peripheral pain. The anti-inflammatory properties of CBG are postulated to result from binding to the CB2 receptor, TRP channels, PPARγ and other targets (De Petrocellis, et al., 2011; Cascio, et al., 2010; Ruhaak, et al., 2011). There is anecdotal human and preclinical evidence for CBG having a benefit in cases of inflammatory bowel diseases including Crohn’s and ulcerative colitis. In a mouse model of colitis, CBG was found to reduce bowel inflammation, nitric oxide production [from increased nitric oxide synthase (iNOS) expression during inflammation] and oxidative stress in intestinal cells (Borrelli, et al., 2013; Pagano, et al., 2021). Similar to CBC, CBG (3 mg/kg and 10 mg/kg i.p.) produces a weak anti-nociceptive effect in mice (Zagzoog, et al., 2020). In one study this effect of CBG was inhibited by the α_2_-adrenergic receptor antagonist yohimbine, suggesting a role of α_2_-adrenergic regulation in CBG analgesia (Cascio, et al., 2010).

Inflammation and oxidative stress are both contributors to neurodegeneration, which is linked to Alzheimer’s and Huntington’s disease as well as Multiple Sclerosis (MS). CBG may protect against both neuroinflammation and oxidative stress, helping to prevent neuronal cell loss (Gugliandolo, et al., 2018). Carrillo-Salinas et al. (2014) examined the effect of the CBG analog VCE-003 on human T-cells and its efficacy in a mouse model of autoimmune MS. When tested *in vitro*, VCE-003 inhibited antigen-induced T-cell proliferation, cell cycle progression and the expression of surface activation markers. VCE-003 also prevented the expression of the pro-inflammatory enzyme iNOS in microglia. In animals, VCE-003 attenuated MS through activating CB2 and PPARγ receptors (Carrillo-Salinas, et al., 2014). CBG was also investigated in a mouse model of Huntington’s disease induced using 3-nitropropionate (3-NP) (Valdeolivas, et al., 2015). Treatment with CBG (10 mg/kg) reduced levels of the pro-inflammatory cytokines IL-6 and tumor necrosis factor α in the 3-NP treated mice. CBG also partially improved motor deficits and preserved striatum neurons in the R2/6 transgenic model of Huntington’s disease (Valdeolivas, et al., 2015).

As noted previously, CBG is effective in suppressing cancer cell growth (Ligresti, et al., 2006). In a murine colon cancer model, CBG was found to promote cancer cell death and inhibit the growth of tumors (Borrelli, et al., 2014). This inhibition was mimicked by TRPM8 channel antagonists (Borrelli, et al., 2014). Additionally, *in vitro* experiments using leukemia cell lines suggest this anti-cancer activity is enhanced when CBG is combined with other cannabinoids such as CBD (Scott, et al., 2013). Clinical studies are currently underway to determine if these results are translatable to treatment in humans. Individuals living with cancer and AIDS commonly experience anorexia and cachexia. CBG represents a non-psychoactive alternative to Δ^9^-THC for treating anorexia since it stimulates appetite and increases food consumption (Brierley, et al., 2017). Interestingly, CBG as part of a whole plant cannabis extract is more potent in stimulating appetite than CBG as an isolate (Brierley, et al., 2017). Thus, these results provide additional evidence that synergism of minor cannabinoids with other components of the cannabis plant may enhance their clinical efficacy (Russo, 2011).

### Cannabinoid Acids

#### Cannabidioloic Acid

CBDA was first isolated in 1955 and its structure elucidated in 1965 (Mechoulam and Gaoni, 1965). CBDA has a low affinity for both the CB1 and CB2 receptors based on ^3^[H]-CP-55,490 displacement assays (Zagzoog, et al., 2020). However, it shows moderate efficacy in inhibiting adenylyl cyclase through these receptors (Navarro, et al., 2020; Zagzoog, et al., 2020). In addition, CBDA is one of several minor cannabinoids (along with THCA and THCV) that functions as allosteric regulators at 5-HT_1a_ receptors (Bolognini, et al., 2013). CBDA enhances 8-OH-DPAT-stimulated (^35^S)GTPγS binding to 5-HT_1a_ receptors expressed in rat brain and CHO cell membranes possibly by binding to an allosteric site on the receptor (Bolognini, et al., 2013). CBDA was reported to be 1,000 times more potent than CBD in stimulating (^35^S)GTPγS binding at the 5-HT_1a_ receptor. Based on *in silico* docking experiments, it was predicted that CBDA, along with the cannabinoids CBGA and CBG, bind to PPARs (D'Aniello, et al., 2019). *In vitro* reporter assays carried out with CHO cells confirmed that all three minor cannabinoids activate PPARα and PPARγ (D'Aniello, et al., 2019).

CBDA produces dose-dependent anti-hyperalgesia and anti-inflammatory effects in a rodent model of carrageenan-induced hind paw inflammation (Rock, et al., 2018). The anti-hyperalgesia effect of CBDA is blocked by AMG9810, an antagonist of the TRPV1 channel. In addition, when combined with Δ^9^-THC, low doses of CBDA are more effective in preventing hyperalgesia and reducing inflammation. CBDA may also produce anti-inflammatory effects *via* cyclooxygenase 2 (COX-2) enzyme inhibition; the same mechanism of action as the NSAID Celecoxib (Takeda, et al., 2008; Ruhaak, et al., 2011). CBDA inhibits the COX-2 enzyme *in vitro* with an EC_50_ of 2 µM and has 9-fold greater selectivity in inhibiting the COX-2 enzyme over the COX-1 enzyme. This selective inhibition is dependent on the presence of the carboxylic acid moiety in the CBDA molecule (Takeda, et al., 2008).

CBDA has anti-nausea effects at low doses in mice that are mediated *via* agonist activity at CNS 5-HT_1A_ receptors (Pertwee, et al., 2018). CBDA was found to be 1000-fold more potent than CBD in reducing nausea-induced conditioned gaping disgust responses (Rock et al., 2020). The drug HU-580, a stable analogue of CBDA that is not metabolized to CBD, also reduces LiCl-induced conditioned gaping (Pertwee, et al., 2018). In addition to suppressing acute nausea, CBDA decreases anticipatory nausea and vomiting which occurs upon re-exposure to a contextual stimulus previously associated with acute nausea (e.g., a chemotherapy session) (Limebeer, et al., 2014). CBDA combined with ondansetron, a commonly used antiemetic drug, enhances ondansetron’s effect when applied at low doses (Rock and Parker, 2013).

Anderson et al. (2019) examined the anti-seizure activity of CBDA using Scn1aRX/+ mouse model of Dravet Syndrome. The Scn1aRX/+ mice develop generalized tonic-clonic seizures in response to elevated body temperature and thus recapitulate the seizures observed in children with Dravet Syndrome. When administered using i.p. injection (10 and 30 mg/kg), CBDA raised the temperature threshold required for seizures in the mice (Anderson, et al., 2019). CBDA also displayed dose-dependent protection in rats against electroshock-induced seizures (Goerl, et al., 2021). While clinical trials have not been reported, CBDA may be more effective than CBD in reducing seizures in humans. According to a patent application by GW Pharmaceuticals, the makers of Epidiolex^®^ (a sublingual spray containing 100 mg of CBD/100 ml of solution), CBDA displays greater bioavailability and potency in treating epilepsy (GW PHARMA LTD, 2015).

#### Cannabigerolic Acid

CBGA is the precursor cannabinoid to THCA, CBDA, and CBCA (see Figure 1). Since CBGA is decarboxylated over time to CBG, it is rarely found in significant amounts in mature cannabis flowers. Thus, harvesting hemp very early yields higher levels of CBGA compared to later in the plant’s life. In addition, some cultivars have increased yields of CBGA through selective breeding to inhibit its transformation into other cannabinoids during the plant’s maturation (Garfinkel, et al., 2021). Similar to CBDA, CBGA displays low affinity for both the CB1 and CB2 receptors (Navarro, et al., 2020). Nonetheless, CBGA is equally as efficacious as Δ^9^-THC in decreasing intracellular cAMP levels though the CB1 receptor (Table 1) (Navarro, et al., 2020). However, unlike Δ^9^-THC, it is not effective in recruiting βarr2 to the CB1 receptor (Navarro, et al., 2020). CBGA has important off target effects including activating PPARs (D'Aniello, et al., 2019). In addition, fractions of *Cannabis sativa* containing high amounts of CBG/CBGA inhibit the aldose reductase enzyme (Smeriglio, et al., 2018).

While less in known about the therapeutic uses of CBGA compared with the other minor cannabinoids, it may play a role in controlling diabetes mellitus and preventing the cardiovascular complications that can accompany Type 2 diabetes (D'Aniello, et al., 2019). Through activation of PPARs, CBGA can improve lipid metabolism and reduce the accumulation of adipose tissue; thus reducing insulin resistance in the Type 2 patient (Gao, et al., 2015). Type 2 diabetes is considered a “coronary artery disease equivalent” and mortality in Type 2 diabetes primarily results from cardiovascular events including acute coronary syndrome (ACS). By inhibiting the enzyme aldose reductase, CBGA improves cardiac glucose metabolism and reduces the risk of ACS (Smeriglio, et al., 2018). Synthetic inhibitors of aldose reductase have severe side effects including elevations in blood liver enzymes from hepatotoxicity, as well as nausea and vomiting. Therefore, plant-derived CBGA offers a promising alternative to these inhibitors.

CBGA may also be beneficial in treating some types of cancer. A cannabis fraction containing high amounts of CBGA was reported to have cytotoxic activity against colon cancer cells (Nallathambi, et al., 2018). Interestingly, synergistic toxic effects were observed when CBGA was given with a cannabis fraction high in THCA. These two fractions also prevented the growth and proliferation of adenomatous colon polyps that are colon cancer precursors. When tested at micromolar concentrations, CBGA was also shown to have cytotoxic actions in human leukemia cancer cell lines (Scott, et al., 2013). In further support of cannabinoid synergism, the IC_50_ for CBGA leukemia cell toxicity was reduced when co-applied with CBD (Scott, et al., 2013; Scott, et al., 2017).

#### Tetrahydrocannabinolic Acid

THCA is a non-psychotropic cannabinoid that is converted to Δ^9^-THC through decarboxylation by exposure to heat (Figure 1 **)**. Since THCA is a precursor to Δ^9^-THC, and because no sample of THCA is completely free of Δ^9^-THC, possession of this cannabinoid could be prosecuted under the U.S. government Federal Analogue Act. THCA displays roughly 60- and 125-fold lower affinity for the CB1 and CB2 receptors compared with Δ^9^-THC (McPartland, et al., 2017). While high concentrations of THCA inhibit forskolin-stimulated increases in cAMP through the CB1 receptor, it produces no inhibition through the CB2 receptor (McPartland, et al., 2017). Nagal et al. (2017) compared the effects of several cannabinoids, including CBDA, CBGA and THCA on PPAR activity. When compared with CBDA and CBGA, THCA has the highest binding affinity for PPARγ (Nadal, et al., 2017). In addition, THCA is more potent than other minor cannabinoids in inducing PPARγ-mediated transcriptional activity.

THCA was recently shown to possess potent anti-inflammatory activity in mice fed a high fat diet (HFD) (Palomares, et al., 2020; Carmona-Hidalgo, et al., 2021). THCA treatment reduced the expression of inflammatory molecules including tumor necrosis factor alpha (TNF-α4) and cytokine interleukin 10 (IL-10) in the HFD mice. This effect was mediated *via* PPARγ stimulation (Palomares, et al., 2020). THCA also improved glucose tolerance and attenuated liver fibrosis in the HFD mice (Carmona-Hidalgo, et al., 2021). Using an *in vitro* COX-1/COX-2 assay it was determined that Δ^9^-THCA inhibits both COX-1 and COX-2 enzymes with a concentration causing 50% inhibition (IC_50_) in the high micromolar range (Ruhaak, et al., 2011). Nallathambi et al. (2017) reported that cannabis fractions containing high amounts of THCA produce anti-inflammatory effects (e.g., reduction in IL-8) in several colon epithelial cell lines and in colon tissue biopsies. Anti-inflammatory effects of THCA were inhibited by treatment with the GPR55 antagonist CID16020046, but not by the CB1 and CB2 receptor antagonist rimonabant and SR144528 (Nallathambi, et al., 2017). In addition to its anti-inflammatory properties, THCA also has anti-nausea and antiemetic properties in mice at doses much lower than Δ^9^-THC (Rock, et al., 2013a). Thus, THCA may present a non-psychotropic alternative to Δ^9^-THC for treating nausea and vomiting.

As discussed previously for CBC and CBG, THCA may also exhibit neuroprotective properties that could be beneficial in the treatment of neurodegenerative diseases. THCA improved neuronal viability through a PPARγ-dependent pathway in an *in vitro* model of Huntington’s disease (Nadal, et al., 2017). THCA also caused an improvement in hind limb dystonia and locomotor activity in mice treated with 3-NPA. These neuroprotective actions of THCA were significantly reduced when mice were co-administered the PPARγ antagonist T0070903. In contrast, THCA had no effects on the survival of dopaminergic neurons in a 1-methyl-4-phenyl pyridinium (MPP^+^) cell culture model of Parkinson’s disease (Moldzio, et al., 2012). Since THCA undergoes decarboxylation to Δ^9^-THC, it is possible that the reported neuroprotective effects of THCA in Huntington’s disease may have resulted from contamination by Δ^9^-THC (Sagredo, et al., 2011). In addition, THCA displays poor brain penetration properties when tested using two vehicles (vegetable oil and Tween 80) (Anderson, et al., 2019); a limitation that could reduce its clinical efficacy.

Anecdotal reports have long suggested that THCA acts as an anticonvulsant. Over 40 years ago Karler and Turkanis reported that THCA (200 mg/kg) reduces seizures in the mouse maximal electroshock test (Karler and Turkanis, 1979). In a more recent mouse study the anticonvulsant effects of THCA were found to vary depending on the seizure model utilized and whether Δ^9^-THC was given along with the THCA (Benson, et al., 2020). When used alone, THCA (2, 30, and 100 mg/kg) was ineffective in the 6-Hz threshold (6-HzT) model of psychomotor seizures, but had anticonvulsant activity when given with Δ^9^-THC. Conversely, THCA used alone or with Δ^9^-THC did not reduce hyperthermia-induced seizures in the Scn1aRX/+ mice model (compared with the protective effects of CBDA described above). More encouraging results were reported with THCA in a clinical study. The frequency and duration of seizures were reduced in four case reports of children using low doses of THCA (0.1–1 mg/kg per day) in conjunction with conventional antiepileptic drugs and full spectrum cannabis (Sulak, et al., 2017). In contrast, Epidiolex^®^ (CBD), which is approved by the U.S. Food and Drug Administration (FDA) for treating epilepsy, is dosed from 5 to 25 mg/kg per day. Thus, THCA may be ten to hundred times more potent in reducing seizures. However, increased doses of THCA did not improve efficacy in this clinical study (Sulak, et al., 2017). Furthermore, formulations of THCA containing high levels of the terpenoid α-linalool were more efficacious than formulations containing low levels of the terpenoid. Thus, other components of the THCA formulation may have accounted for the beneficial effects. Finally, symptoms and seizure activity worsened in one patient after increasing the THCA dose (Sulak, et al., 2017).

### Varinic Cannabinoids

#### Cannabidivarin

CBDV is found in landrace cannabis strains that have relatively high amounts of CBD and low amounts of Δ^9^-THC. Prior to its isolation in 1969, it was assumed that all naturally occurring cannabinoids contained a pentyl side chain, rather than the propyl chain found in CBDV and THCV. CBDV displays low binding affinity for the CB1 and CB2 receptors (Rosenthaler, et al., 2014; Husni, et al., 2014). Consistent with this, high concentrations of CBDV are needed for CB1 receptor stimulation of (^35^S)GTPγS binding, inhibition of cAMP synthesis and recruitment of βarr2 (Husni, et al., 2014; Navarro, et al., 2020; Zagzoog, et al., 2020). Overall, CBDV is a more potent and efficacious agonist at CB2 receptors (Navarro, et al., 2020; Zagzoog, et al., 2020). CBDV displays a similar pharmacological profile for TRP channels as CBN, CBG and THCV; activating TRPV1, TRPV2, TRPV3, TRPV4 and TRPA1 channels while inhibiting the TRPM8 channel (De Petrocellis, et al., 2008; De Petrocellis, et al., 2011). Other important off target sites for CBDV include the de-orphanized receptors GPR55 and GPR6. Binding of CBDV to the GPR55 receptor stimulates ERK1/2 phosphorylation and inhibits LPS-mediated signaling effects occurring through the GPR55 receptor (Anavi-Goffer, et al., 2012). These effects of CBDV are comparable to those of Δ^9^-THC. GPR6 is a constitutively active receptor that couples to G_s_ to stimulate adenylyl cyclase and recruits βarr2. CBDV acts as an inverse agonist at the GPR6 receptor causing significant inhibition of βarr2 recruitment at concentrations of 1 and 10 µM (Laun, et al., 2018).

CBD (marketed as Epidiolex^®^) was approved by the U.S. Food and Drug Administration (FDA) in 2018 for preventing epileptic seizures in Lennox-Gastaut syndrome and Dravet syndrome in children. CBDV, a structural homolog of CBD, possesses anti-epileptic properties when tested in animals and humans. When examined *in vitro* in rat brain slices, CBDV attenuates epileptiform local field potentials induced by 4-amino pyridine (Hill, et al., 2012). *In vivo*, CBDV (200 mg/kg per day) significantly reduces PTZ-induced seizure activity in the rats (Hill, et al., 2012). However, when used alone, CBDV has no effect on pilocarpine-induced seizures, but requires the co-administration of valproate or phenobarbital to be effective. Consistent with this, Amada et al. (2013) reported that CBDV significantly decreases PTZ-induced seizure severity. In addition, CBDV suppresses the expression of several epilepsy-related genes in animals that respond to CBDV anti-epileptic treatment (Amada, et al., 2013). A human trial to assess the efficacy, safety, and tolerability of CBDV in adults with focal seizures was recently conducted by GW Pharmaceuticals, the maker of Epidiolex^®^ (Brodie, et al., 2021). The drug GPW42006 (800 mg b.i.d.), containing CBDV as its major component, reduced the frequency of seizures by 41%. However, similar reductions in focal seizure frequency were observed in the CBDV and placebo (38%) groups. There was also no differences between CBDV and placebo groups for any specific seizure type. Therefore, higher doses and longer durations of treatment of GPW42006 will be needed in future clinical trials to better access the benefits of CBDV.

CBDV has been investigated as a treatment for symptoms associated with autism spectrum disorder (ASD) such as repetitive behaviors, cognitive challenges and issues with communication and social functioning (Mouro, et al., 2019). Mice carrying mutations in the MeCP2 gene and MeCP2 null mice develop Rett Syndrome (RTT), a neurodevelopment disease related to ASD. CBDV treatment (2 mg/kg) was found to rescue both behavioral and phenotypic changes in the RTT mice model (Vigli, et al., 2018). These CBDV effects included improvements in motor coordination, locomotion and brain weight. When administered using a life-long treatment schedule, CBDV also prolonged survival and delayed the appearance of neurological and motor deficits in MeCP2 null mice (Zamberletti, et al., 2019a). CBDV also reversed memory deficits in these mice. Similar behavioral improvements were reported using CBDV in a valproic acid-induced model of ASD (Zamberletti, et al., 2019b).

CBDV may also have utility in the treatment of Duchenne muscular dystrophy (DMD) and in preventing nausea. In a recent study, CBDV was found to improve muscle quality and locomotion and to slow muscle degeneration in male dystrophic mdx mice (Iannotti, et al., 2019). Muscle improvement by CBDV (and CBD) was accompanied by anti-inflammatory and pro-autophagic effects. Both CBDV (200 mg/kg) and THCV (20 mg/kg) are effective in reducing LiCl-induced conditioned gaping in rats (Rock, et al., 2013b). In contrast, CB1 receptor inverse agonists such as SR141716 and AM251 are known to enhance nausea. Thus, CBDV and THCV do not function *in vivo* as CB1 receptor inverse agonists. In conclusion, CBDV has Δ^9^-THC-like, antiemetic effects in rodents consistent with a CB1 receptor agonist, but without the psychotropic activity of Δ^9^-THC.

#### Tetrahydrocannabivarin

THCV is derived from cannabigerovarin acid (CBGVA), one of the two primary minor cannabinoid precursors, the other being CBGA (Figure 1). THCA synthase converts CBGVA to THCVA, which is then decarboxylated to the neutral compound THCV when exposed to heat or light (Hanus, et al., 2016). THCV is typically found in very small amounts in cannabis flowers, though breeders are developing strains with higher concentrations. While THCV binds to both CB1 and CB2 receptors, its pharmacological effects remain controversial. In some studies, THCV has been reported to act as an antagonist/inverse agonist at the CB1 and CB2 receptors. In (^35^S)GTPγS binding assays measured with rodent brain preparations, THCV acts as an antagonist to WIN-55,212-2 (Dennis, et al., 2008). In addition, THCV antagonizes CP-55,940-induced stimulation of (^35^S)GTPγS binding in rodent brain and CHO cell membranes (Thomas, et al., 2005). In contrast, more recent studies using CHO cells have demonstrated that THCV functions as a partial agonist at the cannabinoid receptors to inhibit cAMP formation and to stimulate βarr2 recruitment (Zagzoog, et al., 2020). Computational docking experiments revealed that THCV interacts with the same residues as Δ^9^-THC in the orthosteric site of the CB1 receptor (Jung, et al., 2018). However, the pentyl side chain of Δ^9^-THC protrudes into a sub-pocket of the binding site. THCV containing a propyl side chain does not have this interaction (Jung, et al., 2018). This difference and the distinct binding energies of the two ligands might account for the higher affinity of Δ^9^-THC for the CB1 receptor.

As with other minor cannabinoids, THCV has off target actions at TRP channels and 5-HT_1A_ receptors. THCV acts as an agonist at TRPV1-4 and TRPA1 channels, while acting as an antagonist at the TRPM8 channel (De Petrocellis, et al., 2011). THCV (at 100 nM) was found to enhance (H^3^)-8-OH-DPAT binding to the 5-HT_1A_ receptor and to increase the potency of 8-OH-DPAT-stimulated (^35^S)GTPγS binding to cell membranes (Cascio, et al., 2015). Thus, both THCV and CBDA appear to function as positive allosteric regulators of the 5-HT_1A_ receptor.

THCV has been shown to reduce inflammation and inflammatory pain in mice. THCV attenuated signs of inflammation induced by intraplantar injection of carrageenan in mouse hind paws and reduced hyperalgesia from formalin hind paw injection (Bolognini, et al., 2010). Surprisingly, the ability of THCV to relieve formalin-induced hyperalgesia was significantly attenuated by both the CB1 receptor-selective antagonist rimonabant, and CB2 receptor-selective antagonist SR144528 (Bolognini, et al., 2010). Thus, when tested *in vivo*, THCV exhibits both CB1 and CB2 receptor agonist activity. These anti-inflammatory actions of THCV were further supported by *in vitro* experiments using peritoneal-derived macrophages (Romano, et al., 2016). THCV was found to suppress inflammatory pathways by down-regulating LPS-induced expression of iNOS, COX-2 and interleukin 1β. THCV mediated suppression of nitrite production (from nitric oxide) was prevented by pretreatment of the macrophages with SR144528.

THCV has also shown promise as an anti-epileptic agent and in the treatment of neurodegeneration in Parkinson’s disease. Hill et al. (2010) used an extracellular multi-electrode array (MEA) assay to study the effects of THCV on spontaneous epileptiform bursting in rat brain slices. Pretreatment of the brain slices with THCV (20 µM) reduced burst complex incidence and the amplitude and frequency of paroxysmal depolarizing shifts (Hill, et al., 2010). In addition, in rats treated *in vivo* with pentylenetetrazole (PTZ) to induce seizures, THCV (0.25 mg/kg) reduced the incidence of seizures. Approximately 33% of animals treated with THCV exhibited a complete absence of PTZ seizures (compared with 13% of control treated rats). Garcia et al. (2011) reported that THCV reduces slow motor movements in rats with 6-hydroxydopamine (6-OHDA)-induced Parkinson’s disease. In addition, 2 weeks of treatment with THCV reduced microglial activation and preserved nigrostriatal dopaminergic neurons in the 6-OHDA Parkinson’s model (García, et al., 2011). Thus, THCV may be a useful treatment for Parkinson’s disease by preventing neuronal degradation and alleviating associated symptoms.

THCV regulates blood glucose levels suggesting it might be useful in weight reduction and treating diabetes. In mice with dietary-induced obesity (DIO), THCV improved fasting plasma glucose and glucose tolerance in a dose-dependent manner (Wargent, et al., 2013). In addition, THCV increased insulin sensitivity in genetically (ob/ob) obese mice. While THCV increased energy expenditure in the DIO and ob/ob mice, it did not reduce food intake or overall body weight (Wargent, et al., 2013). This contrasts with a previous study where intraperitoneal administration of THCV caused hypophagia and weight loss in rodents (Riedel, et al., 2009). Importantly, a clinical trial evaluated the effects of THCV and CBD on 62 subjects with type 2 diabetes (Jadoon, et al., 2016). Although THCV had no effect on plasma HDL levels, it significantly decreased fasting plasma glucose levels and improved pancreatic β-cell function in the type 2 patients. In addition to these findings, THCV was reported to increase the response to aversive stimuli in humans in regions of the brain (amygdala, insula and mid orbitofrontal cortex) involved in food aversion (Tudge, et al., 2015). This suggests that THCV may aid in appetite suppression and weight loss without the side effects (depression, anxiety, etc.,) caused by the CB1 receptor antagonist rimonabant (Mitchell and Morris, 2007).

Various minor cannabinoids including THCV, CBC, CBG and CBDV have shown promise in the treatment of skin disorders and are being investigated for the treatment of atopic dermatitis, psoriasis, scleroderma, acne hair growth and pigmentation disorders, keratin diseases, skin tumors, and pruritus (Tubaro, et al., 2010; Oláh, et al., 2016; Tóth, et al., 2019). It is postulated that these cannabinoids produce anti-acne effects by regulating homeostatic sebaceous lipogenesis and by exerting anti-proliferative and anti-inflammatory actions. *In vitro* experiments have shown that THCV inhibits the proliferation of human SZ95 sebocytes (Oláh, et al., 2016). This anti-proliferative effect of THCV occurs through a CBD-like mechanism of action; increasing intracellular Ca^2+^ and stimulating ERK1/2 following TRPV4 channel activation. In addition, THCV exhibits powerful anti-inflammatory properties by reducing levels of arachidonic acid (AA), needed for lipogenesis (Oláh, et al., 2016). THCV also suppresses lipid synthesis in the sebaceous glands, providing relief to acne sufferers whose condition is triggered by excessive oil production (Oláh, et al., 2016; Tóth, et al., 2019). In conclusion, THCV and other minor cannabinoids will continue to be evaluated for the management of acne.

### Other Minor Cannabinoids

Cannabitriol (CBT) and Δ^8^-tetrahydrocannabinol (Δ^8^-THC) are two rare cannabinoids that are gaining commercial popularity. Cannabitriol (CBT) was first isolated by Obata and Ishikawa, but its structure was not fully determined until 1977 (Obata and Ishikawa, 1966; ElSohly, et al., 1977). Although the pharmacology of CBT is largely unknown, recent virtual screening analysis of the estrogen receptor α (ER-α) indicate that CBT represents a novel estrogen antagonist that might be used for the prevention and treatment of breast cancer (Kikiowo, et al., 2021). Δ^8^-THC is an isomer of Δ^9^-THC that contains a double bond between carbon atoms 8 and 9. Unlike Δ^9^-THC, Δ^8^-THC is legally available in the U.S. through cannabis suppliers. The U.S. 2018 Farm Bill legalized cannabinoids such as CBD that are isolated from hemp. Since Δ^8^-THC can be derived from CBD, it is currently considered a legal natural product. While Δ^8^-THC displays roughly similar binding affinities as Δ^9^-THC to the CB1 and CB2 receptors (Husni, et al., 2014), preclinical results suggest that it is less potent in producing euphoric, anti-emetic and appetite-stimulating effects (Järbe and Henriksson, 1973; Hine, et al., 1977).

## Conclusion

**FIGURE 3:**
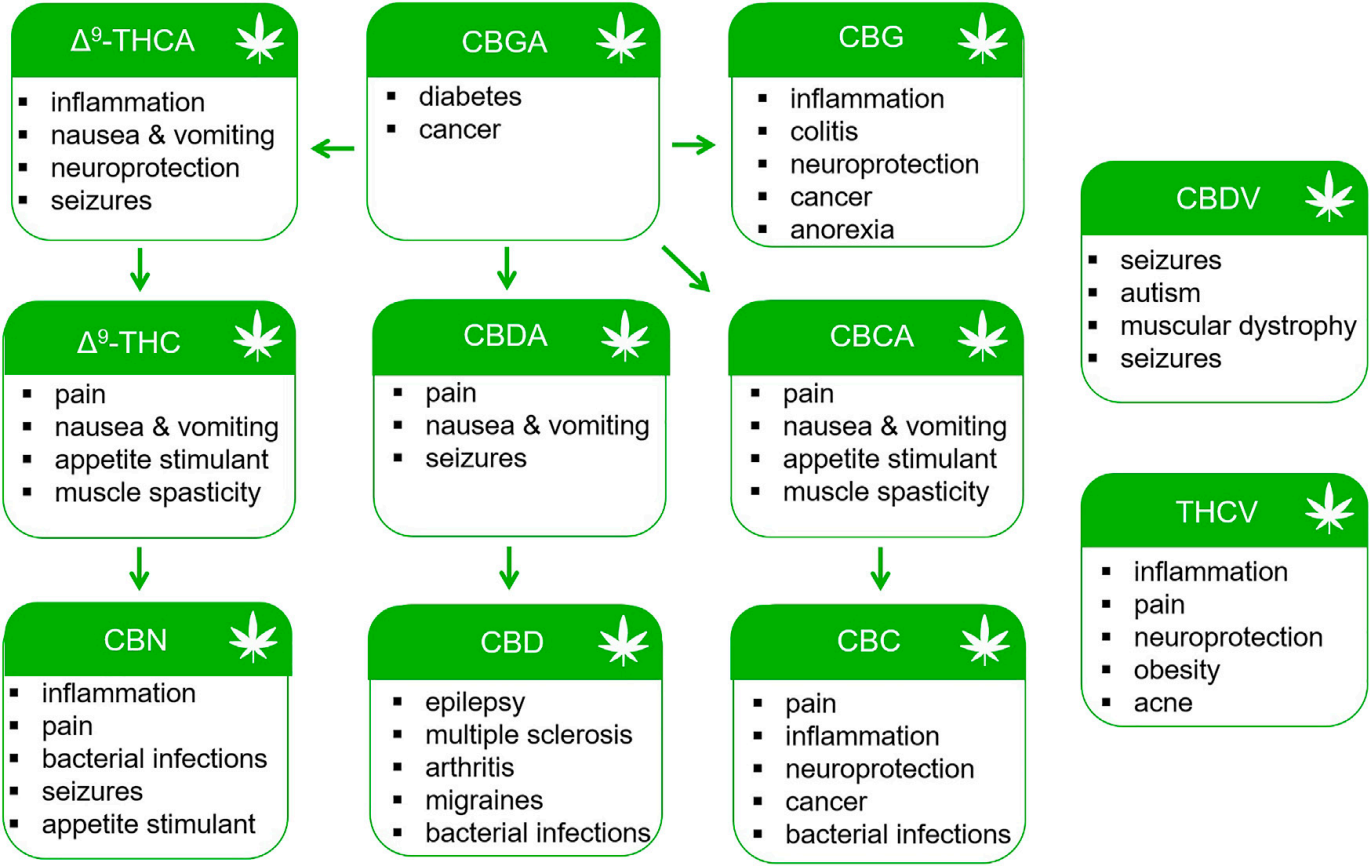
Potential therapeutic uses of phytocannabinoids.

Preclinical data and early clinical studies support the continued investigation of phytocannabinoids for the treatment of pain, inflammation, neurodegeneration, cancer and other disorders (Figure 3). Natural products have historically been valuable sources of novel compounds developed into pharmaceuticals. Such was the case with the isolation of salicin from the bark of the Willow tree and the subsequent synthesis of aspirin. Δ^9^-THC (Dronabinol) is currently approved by the U.S. FDA for the treatment of nausea associated with cancer chemotherapy and as an appetite stimulant for patients with AIDS (Romero-Sandoval, et al., 2018; Fraguas-Sánchez and Torres-Suárez, 2018). Nabiximols (Sativex^®^) containing a mixture of Δ^9^-THC and CBD from the cannabis plant is approved in Canada and Europe for the treatment of MS spasticity (Fraguas-Sánchez and Torres-Suárez, 2018). It is also indicated for the treatment of neuropathic pain in MS and for pain relief in patients with advanced cancer (Fraguas-Sánchez and Torres-Suárez, 2018). However, use of Δ^9^-THC is associated with acute psychotropic effects including euphoria, sedation, anxiety, cognitive impairment, and in some patients, paranoia and hallucinations. Minor cannabinoids and their chemical homologs offer the potential medicinal benefits of Δ^9^-THC without adverse effects. Recently, Δ^9^-tetrahydrocannabiphorol (Δ^9^-THCP) and cannabidihexol (CBDH), homologs of Δ^9^-THC and CBD, were synthesized and shown to produce anti-nociceptive effects in mice at doses comparable to Δ^9^-THC (Citti, et al., 2019; Linciano, et al., 2020). Future studies will need to evaluate the risk versus benefit of these and other minor cannabinoids when compared to Δ^9^-THC and traditional analgesic drugs.

In addition to the CB1/CB2 receptors and “off target” binding sites described in this review, minor cannabinoids may bring about their pharmacological effects by interacting with other receptors and ion channels. Along with GPR55 and GPR18, de-orphanized receptors including GPR3, GPR6 and GPR12 are emerging as possible targets for minor cannabinoids (Laun and Song, 2017; Brown, et al., 2017). These receptors are highly expressed in neuronal tissues and are postulated to participate in neuroprotection, anti-nociception and brain development. Although the affinity of these receptors for minor cannabinoids has not yet been examined, CBD is known to function as an inverse agonist at all three receptors. However, it is unclear whether CBD binds to an orthostatic site on the receptor or if it modifies receptor activity *via* an allosteric site. While TRP channel agonism/antagonism provides a major mechanism of action for many of the minor cannabinoids, voltage-gated ion channels, such Na^+^ and Ca^2+^ channels are also regulated by cannabinoids. When tested in parathyroid cells, the synthetic cannabinoid WIN 55,212-2 and the endocannabinoid 2-AG reduce the peak Na^+^ current and shift the voltage-dependence of Na^+^ channel inactivation to more negative membrane potentials (Okada, et al., 2005). In addition, when applied at low micromolar concentrations, CBD inhibits the Na^+^ current in heterologous cells expressing various Na^+^ channel subunits (Na_V_1.1, Na_V_1.3 Na_V_1.6, etc.,) (Ghovanloo, et al., 2018). CBD also inhibits T-type Ca^2+^ channels (Ca_V_3.x) in mouse sensory neurons (Ross, et al., 2008). Whether CBD acts directly to regulate the conduction of the Na^+^ and Ca^2+^ channels, or acts indirectly to alter the properties of the cell lipid membrane will require further investigation.

Advances in the bioengineering of cannabinoid synthesis enzymes in yeast and other microbial systems should expand the production of both natural and novel minor cannabinoids (Luo, et al., 2019). The ability to combine these cannabinoids with terpenes, flavonoids, polyphenols and other cannabis-based chemicals could create countless possibilities in the era of personalized healthcare. It is predicted that new cannabinoid products might be formulated to meet the therapeutic needs of different demographic groups and could be available in numerous delivery systems including topical creams, tablets, transdermal patches, vaporizers and more. Women represent one demographic group where cannabinoids could offer a variety of health care benefits. Cannabinoid receptors are ubiquitously distributed in reproductive tissues and AEA and the FAAH enzyme are found in the ovaries, oviducts and endometrium (Maia, et al., 2020). Cannabinoid-based suppositories containing Δ^9^-THC and CBD are already available for relieving menstrual cramps, and as drug discovery progresses, natural and unnatural cannabinoids may prove effective for reproductive system issues, from endometriosis and fibroids to perimenopause symptoms. Of course, the effectiveness of these cannabis products must first be confirmed through large, randomized and controlled clinical trials. Much of our current knowledge of the medicinal benefits of minor cannabinoids has come from subjective and anecdotal patient reporting, rather than through rigorous clinical trials. In order to move forward, researchers, clinicians and regulatory officials will need to work together to ensure that phytocannabinoid products meet the necessary therapeutic and safety standards.
